# Supplementary material for: Salvia plebeia R.Br. and Rosmarinic Acid Attenuate Dexamethasone-Induced Muscle Atrophy in C2C12 Myotubes
Source: Int J Mol Sci. 2023 Jan 18;24(3):1876. doi: 10.3390/ijms24031876 (PMC9915874; doi:10.3390/ijms24031876)
Supplement: Supplementary file 1 [file ijms-24-01876-s001.zip › ijms-2105815-supplementary.pdf]

# *Salvia plebeia* R.Br. and Rosmarinic Acid Attenuate Dexamethasone-Induced Muscle Atrophy in C2C12 Myotubes

**Jae-Yong Kim †, Hye Mi Kim †, Ji Hoon Kim, Shuo Guo, Do Hyun Lee, Gyu Min Lim, Wondong Kim * and Chul Young Kim ***

College of Pharmacy and Institute of Pharmaceutical Science and Technology, Hanyang University, Ansan 426-791, Gyeonggi-do, Republic of Korea

* Correspondence: wdongkim@hanyang.ac.kr (W.K.); chulykim@hanyang.ac.kr (C.Y.K.); Tel.: +82-31-400-5817 (W.K.); +82-31-400-5809 (C.Y.K.); Fax: +82-31-400-5958 (C.Y.K.)

† These authors contributed equally to this work.

**Supplementary Figure S1**.

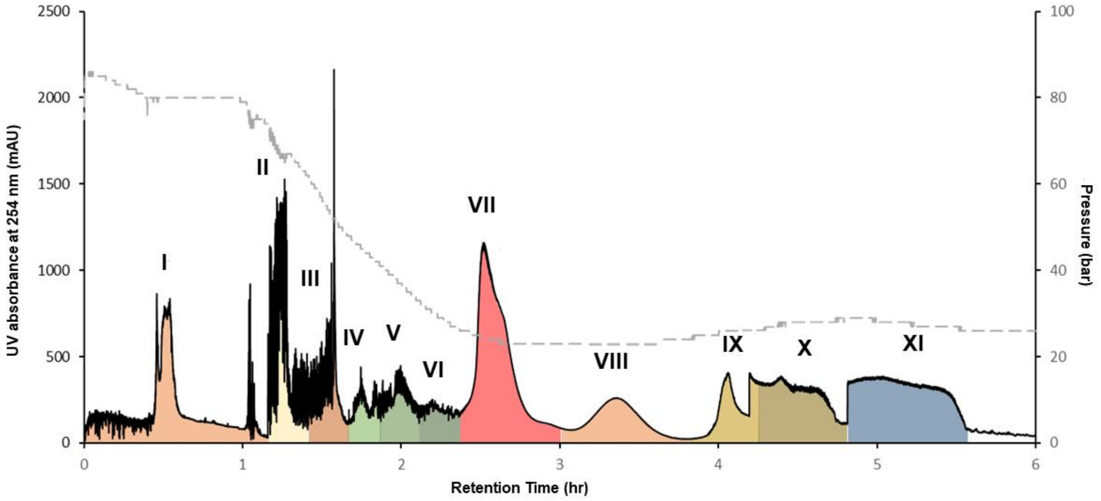


**Figure S1. Gradient mode CPC chromatogram of EtOH extract of *Salvia plebeia* R. Br.**

**Table S1. The CPC fractions weight of SPR and calculated concentrations.**

| Fractions | Weight (mg) | Weight ratio (%) | Treatment (μg/ml) |
|---|---|---|---|
| I | 167.4 | 16.7 | 1.67 |
| II | 23.4 | 2.3 | 0.23 |
| III | 107.4 | 10.7 | 1.07 |
| IV | 61.8 | 6.2 | 0.62 |
| V | 74.1 | 7.4 | 0.74 |
| VI | 40.2 | 4.0 | 0.40 |
| VII | 141.9 | 14.1 | 1.41 |
| VIII | 47.1 | 4.7 | 0.47 |
| IX | 32.6 | 3.2 | 0.32 |
| X | 29.6 | 2.9 | 0.29 |
| XI | 29.9 | 3.0 | 0.30 |
| R (rotor residue) | 249.2 | 24.8 | 2.48 |
| SUM | 1004.6 | 100 | 10 |

**Supplementary Figure S2**.

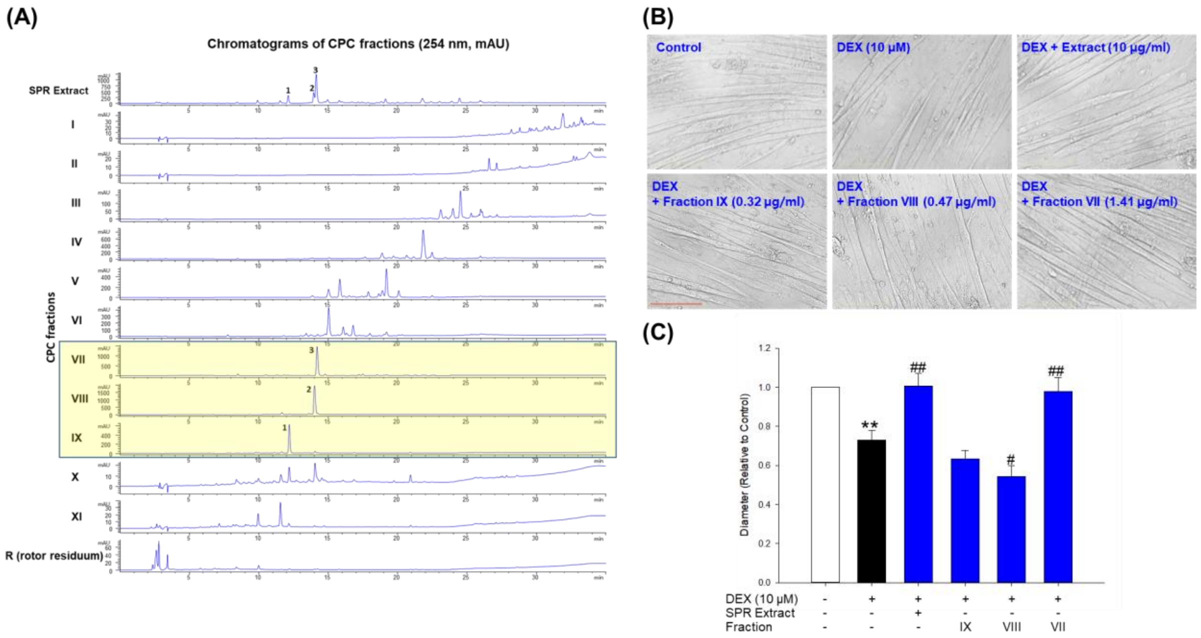


**Figure S2. HPLC chromatogram of *Salvia plebeia* R. Br. and its CPC fractions and their effects on DEX-mediated atrophy in C2C12 myotubes.** (A) The mobile phase consisted of acetonitrile (0.1 % formic acid, solvent A) and water (0.1 % formic acid, solvent B) in a gradient mode: 0 min, 10 % A; 5 min, 20 % A; 20 min, 40 % A; 30 min, 95 % A; 35 min, 95 % A. The flow rate was 1 mL/min and the injection volume was 10 μL. The photodiode array (PDA) detector employed a UV spectrum over a range of 210 to 400 nm and the chromatogram of the effluents was recorded at 254 nm. (B) Representative photographs of C2C12 myotubes for control, DEX, DEX + fractions IX, DEX + fractions VIII, and DEX + fractions VII treatments (scale bar = 250 μm ). (C) Relative changes in myotube diameters were observed from randomly selected fields and were quantified using the Image J program. These results are presented as means ± SD of three independent experiments: $^{**}p < 0.01$ vs. control; $^{\#}p < 0.05$, $^{\#\#}p < 0.01$ vs. DEX.

**Supplementary Figure S3**.

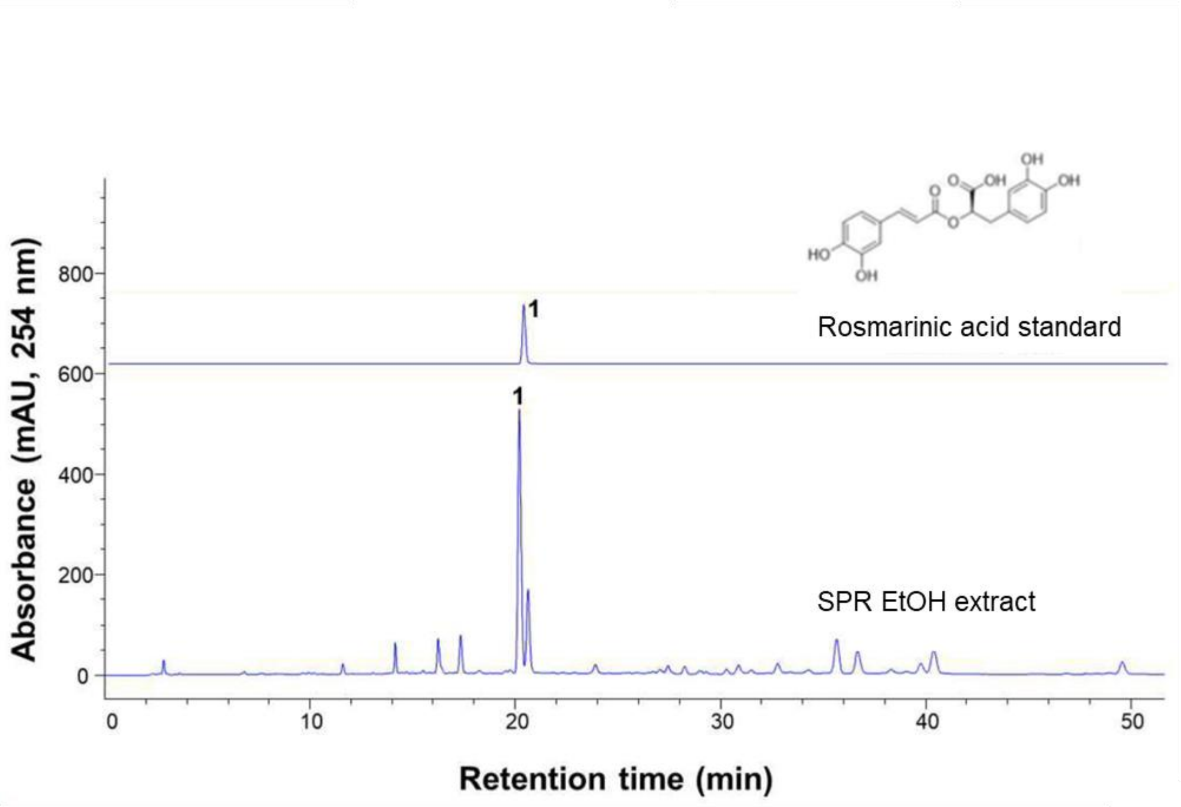


**Figure S3. HPLC-UV/PDA chromatogram of the EtOH extract of *Salvia plebeia* R. Br. and rosmarinic acid standard.** The acetonitrile (0.1 % formic acid, solvent A) and water (0.1 % formic acid, solvent B) were used as the mobile phase in a gradient mode: 0 min, 5 % A; 10 min, 20 % A; 55 min, 40 % A and the stationary phase was used in the C18 column. The photodiode array (PDA) detector employed a UV spectrum over a range of 210 to 400 nm and the wavelength of 254 nm was chosen for the suitable detection because the resolution and baseline are much better than other wavelengths in the chromatogram.

**Supplementary Figure S4**.

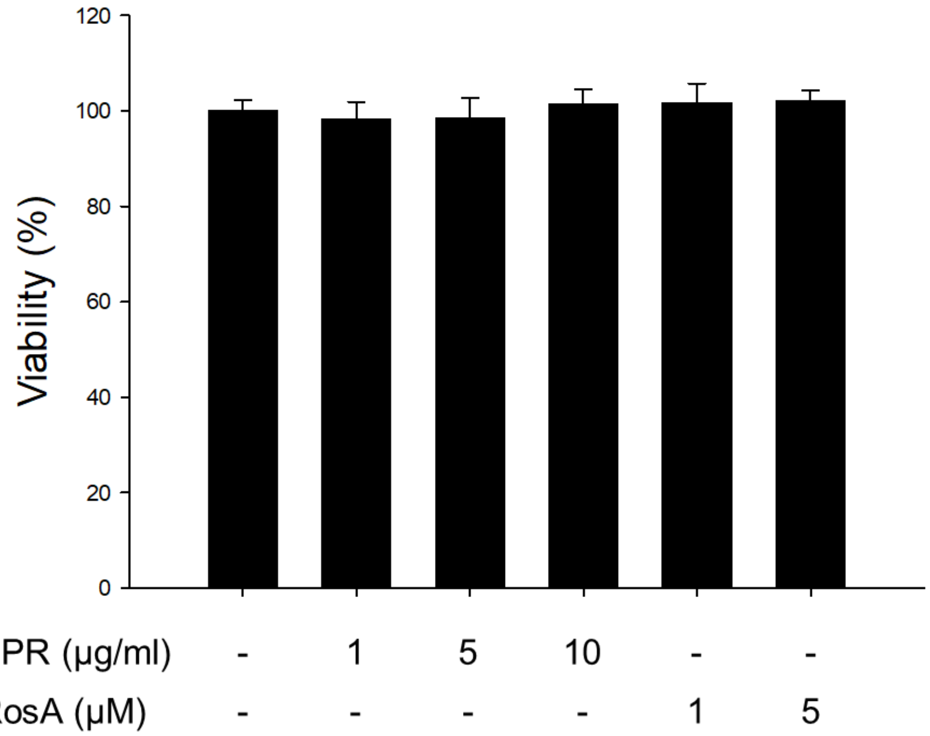


**Figure S4. C2C12 myotubes proliferation in the presence of the SPR and RosA.** C2C12 myotubes were cultured in differentiation media supplemented with the SPR (1, 5, and 10 µg/mL) or RosA (1 and 5 µM) for 24 h. The myotubes proliferations were determined by CCK-8 assay.
